# A Decomposition-Based Multi-Objective Flying Foxes Optimization Algorithm and Its Applications

**DOI:** 10.3390/biomimetics9070417

**Published:** 2024-07-07

**Authors:** Chen Zhang, Ziyun Song, Yufei Yang, Changsheng Zhang, Ying Guo

**Affiliations:** 1 Software College, Northeastern University, Shenyang 110169, China; 2 College of Computer Science and Engineering, Ningxia Institute of Science and Technology, Shizuishan 753000, China

**Keywords:** flying foxes optimization (FFO) algorithm, MOEA/D, multi-objective optimization problems, bio-inspired algorithms, real-world applications

## Abstract

The flying foxes optimization (FFO) algorithm stimulated by the strategy used by flying foxes for subsistence in heat wave environments has shown good performance in the single-objective domain. Aiming to explore the effectiveness and benefits of the subsistence strategy used by flying foxes in solving optimization challenges involving multiple objectives, this research proposes a decomposition-based multi-objective flying foxes optimization algorithm (MOEA/D-FFO). It exhibits a great population management strategy, which mainly includes the following features. (1) In order to improve the exploration effectiveness of the flying fox population, a new offspring generation mechanism is introduced to improve the efficiency of exploration of peripheral space by flying fox populations. (2) A new population updating approach is proposed to adjust the neighbor matrices to the corresponding flying fox individuals using the new offspring, with the aim of enhancing the rate of convergence in the population. Through comparison experiments with classical algorithms (MOEA/D, NSGA-II, IBEA) and cutting-edge algorithms (MOEA/D-DYTS, MOEA/D-UR), MOEA/D-FFO achieves more than 11 best results. In addition, the experimental results under different population sizes show that the proposed algorithm is highly adaptable and has good application prospects in optimization problems for engineering applications.

## 1. Introduction

Multi-objective optimization problems (MOPs) [1,2] are an important branch of the optimization field aiming to expedite the optimization of multiple objective functions concurrently. These problems are widely used across diverse sectors like electrical and power systems, scheduling, wireless and network systems, robotics, classification and forecasting, cloud computing, image processing, environment engineering domains, and many others [3,4]. The optimization problems involved in these applications are complex and high-dimensional, which puts higher demands on the effectiveness of solving application problems. A multi-objective optimization problem is identified as shown in Formula (1).

(1) $$\left\{\begin{array}{c} minF(X)=(f_{1}(X),f_{2}(X),\ldots ,f_{m}(X){)}^{T}\\ subject\;to:X=(x_{1},x_{2},\ldots ,x_{n})\epsilon \Omega \end{array}\right.$$

In the domain of decision making, represented by *Ω*, a function *F* assigns each element to a vector within the *m*-dimensional real-valued space of outcomes, denoted by *R_m_*, where each dimension corresponds to an objective function. For a given *n*-dimensional decision variable *X = (x*_1_, *x*_2_, …, *x_n_) ϵ Ω*, *X* represents a solution for the MOP objective. Suppose that *X_A_*, *X_B_ ϵ Ω* are two solutions to a MOP if and only if the conditions are satisfied in Formula (2) and there exists *j ϵ {1, …, m}* such that *X_A_* dominates *X_B_* when *f_j_(X_A_) < f_j_(X_B_)*, written as *X_A_* ≺ *X_B_*.

(2) $$f_{i}\left(X_{A}\right)\leq \;f_{i}\left(X_{B}\right),\;\forall i\;\epsilon \;\{1,\ldots ,m\}$$

Within the framework defined, a solution X^*^ achieves Pareto optimality if it is not dominated by any alternative within *Ω*. The set comprising all such Pareto-optimal solutions is specified in Formula (3).

(3) $$PS=\left\{X^{*}\;\right|\neg \exists X\;\epsilon \;Ω\;,\;X≺X^{*}\;\}$$

Aiming to tackle MOPs, the researchers proposed a multi-objective evolutionary algorithm and widely applied it to solve real-world problems [5,6,7], such as portfolio optimization, supply chain management, highway construction processes, argi-technologies processes, logistics and transportation systems, etc. These applications provide a continuous source of vitality for the research of multi-objective evolutionary algorithms. Among them, a multi-objective evolutionary algorithm based on decomposition (MOEA/D) [8], as a multi-objective evolutionary algorithm based on the traditional aggregation method, is efficient and versatile for problem decomposition. There are three main methods for decomposing multi-objective problems: the boundary intersection (BI) approach, the weighted sum approach, and the Tchebycheff approach. MOEA/D is fundamentally designed to decompose complex multi-objective optimization challenges into multiple scalarized optimization tasks. This method seeks to approximate the Pareto front (PF) [9,10,11] through evolutionary strategies and cooperative interactions between these individual tasks. When a MOP is decomposed into *N* subproblems, each subproblem is associated with weight vectors *λ* that are uniformly distributed and satisfy Formula (4).

(4) $$\lambda =(\lambda_{1},\lambda_{2},\ldots ,\lambda_{N})\;\lambda_{1}+\lambda_{2}+\ldots +\lambda_{N}=1$$

The No Free Lunch (NFL) theorem [12] has led to the development of a large number of new algorithms. Among them, MOEA/D is regarded as the underlying design model, which has been extended by many researchers into a variety of other versions [13,14,15,16]. By embedding different optimizers into MOEA/D, the possibilities of using different operators for efficiently solving MOPs in a decomposition-based framework are explored. With the progress in computational intelligence, there is a growing focus on swarm intelligent optimization algorithms, which are well-suited to dealing with complex optimization problems and finding high-quality solution sets in a shorter period of time. Researchers have embedded swarm intelligence optimization algorithms as optimizers into MOEA/D to further improve the solution efficiency and quality when solving different types of MOPs.

Recently, the flying foxes optimization (FFO) algorithm [17] demonstrated excellent performance in the exploration of single-objective problems. The FFO algorithm employs a double penalty mechanism for the flying fox operator. For the flying fox operators whose search areas are too remote and have poor search results, a death mechanism is implemented to prevent individuals from carrying out meaningless migration operations in the same place and stop the population from moving in the wrong direction. For the individuals gathering near the optimal solution on a local scale, the suffocation strategy is implemented to discourage the population from converging towards the local optima because of rapid convergence and an inability to continue to search for the global optimal solution, which greatly improves the algorithm’s rate of convergence and the accuracy of the solution. The FFO algorithm has demonstrated excellent results in real-world engineering problems, such as the flow-shop scheduling problem (FSP) [18,19], the minimum spanning tree (MST) problem [20], and the p-hub location allocation (HLA) problem [21], through its well-designed survival strategy and implementation.

Given the successful application of swarm-intelligence-based optimization algorithms in MOEA/D and the robust results of the flying foxes’ survival strategy within the FFO algorithm for single-objective optimization, this study introduces a novel multi-objective evolutionary algorithm. This algorithm aims to provide fresh perspectives for solving MOPs. It uses the traditional MOEA/D algorithm as a framework and incorporates the survival strategy of the FFO algorithm, which is called a decomposition-based multi-objective flying foxes optimization algorithm (MOEA/D-FFO). The main research outlined in this paper includes the following:

(1) A new, efficient algorithm is provided for solving MOPs. By introducing the flying foxes’ survival strategy with a penalty mechanism into MOEA/D, the FFO algorithm is employed for the inaugural time in addressing multi-objective optimization challenges, and MOEA/D-FFO is proposed. We also further explore the performance of MOEA/D-FFO in solving MOPs.
(2) In order to make the survival strategy of the FFO algorithm more suitable for solving MOPs, three improvements are made in conjunction with the original flying foxes’ survival strategy. Firstly, the position calculation strategy of the flying fox individual is designed and implemented, where the value derived from each single objective function generated after the decomposition of the multi-objective problem is multiplied and summed with the corresponding weight vector of the flying fox individual. Second, in terms of the iterative approach, a new offspring generation mechanism is introduced. By adjusting the over-distance death mechanism in the flying foxes’ survival strategy, individuals that are too far away from the optimal solution in the population can explore other possible solutions to the MOPs, which helps to preserve the variety within the population. Thirdly, in terms of population delineation, a new population renewal mechanism is proposed. Instead of iterating all flying foxes as a whole, individual flying foxes and their corresponding neighbors are treated as a small population, thus generating multiple flying fox populations for iteration. This improvement speeds up the convergence of populations.
(3) Compared to the cutting-edge multi-objective evolutionary algorithms (MOEAs), the algorithm proposed in this paper is subjected to a series of experiments on two multi-objective test suites and three real-world applications, and the experimental data illustrate its effectiveness in solving MOPs.

This paper is structured in the following manner. In Section 2, the focus is on the introduction of the multi-objective evolutionary algorithm. It details the effectiveness of the MOEA/D framework along with the survival strategy of the FFO algorithm. Section 3 details the architectural underpinnings of the MOEA/D-FFO and explores the principal enhancements introduced. In Section 4, we compare the performance of the MOEA/D-FFO algorithm with alternative methods through the application of the DTLZ and ZDT benchmark series. Additionally, its performance is assessed through the application to three real-world MOPs. Section 5 concludes this paper by summarizing the advantages of MOEA/D-FFO and proposing future research directions.

## 2. Related Work

### 2.1. Multi-Objective Evolutionary Algorithms

In the process of continuous development and maturation of evolutionary algorithms, numerous classical multi-objective evolutionary algorithms have been developed, and they continue to be widely applied due to their outstanding performance. For example, Deb et al. [22] proposed the non-dominated sorting genetic algorithm II (NSGA-II), which employs the concepts of non-dominated sorting and crowding distance to organize the population by categorizing individuals into various non-dominated levels and then utilizes crowding distance to arrange individuals within the same level, ensuring population diversity within the algorithm. For the multi-objective evolutionary algorithm utilizing metric-driven search, Zitziler et al. [23] proposed that the core of this algorithm lies in the concept of initially formulating the optimization objective through a binary performance metric, followed by its direct integration into the selection process. This means that in this algorithm this metric can be combined with any metrics to be adjusted according to the specific needs, and in it no further diversity preservation mechanisms are necessary. Research conducted on various continuous and discrete benchmark problems has demonstrated the significant enhancements of NSGA-II and the strength Pareto evolutionary algorithm (SPEA2) [24] through the implementation of the indicator-based evolutionary algorithm (IBEA). Significant progress in multi-objective evolutionary algorithms has been achieved through intensive research into complex multi-objective optimization issues. Developed by Zhang et al., MOEA/D [8] has transformed the approach to multi-objective problems by segmenting them into several single-objective tasks. This method significantly boosts the effectiveness of multi-objective evolutionary algorithms. By converting multi-objective optimization challenges into a series of single-objective subproblems, MOEA/D facilitates their simultaneous optimization using evolutionary algorithms. It enhances this process by incorporating data from adjacent subproblems, which not only preserves the diversity of solutions through its decomposition technique but also reduces the likelihood of the population converging towards local optima due to the integration of nearby problem insights.

The principles of decomposition in MOEA/D offer a straightforward and efficient approach for enhancing multi-objective evolutionary algorithms, particularly in addressing certain challenges, such as fitness alignment and diversity preservation. For instance, Liangjun Ke et al. introduced MOEA/D-ACO, amalgamating the ant colony algorithm with MOEA/D and integrating the ant colony algorithm’s emulation of ants’ path selection behavior based on pheromone concentration [25]. Similarly, Yutao Qi et al. devised MOEA/D-AWA, incorporating an adaptive weight adjustment mechanism [26]. Zhang et al. presented MOEA/D-EGO tailored to addressing complex multi-objective optimization tasks [27]. Leveraging the iterative population strategy from single-objective optimization algorithms to expand MOEA/D constitutes a prevalent approach in contemporary multi-objective algorithm research. In this study, we adopt the established multi-objective framework of MOEA/D and synergize it with the flying foxes’ survival strategy, resulting in the development of an innovative multi-objective evolutionary algorithm. Our aim is to further explore efficient algorithms for solving MOPs.

### 2.2. FFO Algorithm

The FFO algorithm is able to better adapt to complex and changing environments and find better solutions in optimization problems by simulating the survival strategy of flying foxes. Compared with current popular metaheuristic algorithms [28,29], especially when dealing with unimodal, multimodal, and fixed-dimension objective functions and solving real-world problems, the FFO algorithm shows a more competitive convergence speed and superior local and global exploration functionalities. The survival strategy of the FFO algorithm mainly consists of the migratory behaviors of the flying foxes in the hot region and the suffocation and death mechanisms of the flying foxes, described as follows.

(1) Movement of Flying Foxes

In the case where the flying fox individual *i* is far from the optimal solution within the population, but the flying fox still has the strength to fly to the position of the ideal solution in the population, the flying fox will fly to the optimal solution to avoid overheating and dying, utilizing Formula (5) to migrate.

(5) $$x_{i,j}^{t+1}=x_{i,j}^{t}+\alpha *rand\left(cool_{i,j}-x_{i,j}^{t}\right)$$

where $x_{i,j}^{t}$ represents the *j*-th element of the flying fox individual *i* in the *t*-th iteration and *cool_i,j_* represents the *j*-th element’s current location of the optimal individual in the population corresponding to individual *i*. α is a positive attraction constant, *rand*~U(0,1).

When a flying fox individual is closer to the optimal solution currently found within the population, it searches for other nearby regions to avoid crowding and suffocation, and it migrates through Formulas (6) and (7).

(6) $${nx}_{i,j}^{t+1}=x_{i,j}^{t}+{rand}_{1,j}*\left({cool}_{i,j}-x_{i,j}^{t}\right)+{rand}_{2,j}*\left(x_{R_{1},j}^{t}-x_{R_{2,}j}^{t}\;\right)$$

(7) $$x_{i,j}^{t+1}=\left\{\begin{array}{c} {nx}_{i,j}^{t+1},\;\;if\;j=k\;or\;{rand}_{3,j}\geq pa\\ x_{i,j}^{t},\;\;otherwise \end{array}\right.$$

Provide that *rand* is uniformly distributed between 0 and 1, which means *rand*_1,*j*_, *rand*_2,*j*_, and *rand_3_*_,*j*_ are randomly generated numbers between 0 and 1. *pa* denotes a probability constant. $x_{R_{1},j}^{t}$ and $x_{R_{2,}j}^{t}$ are two randomly selected flying fox individuals’ *j*-th elements within all of the current population. *k* is a number chosen at random from (1, 2, …, *d*) to guarantee the existence of a minimum of one element of the ${nx}_{i,j}^{t+1}$ individuals is selected to form $x_{i,j}^{t+1}$ so that the newly generated flying fox individual will not be the same as the original flying fox individual *i*, ensuring that the newly generated flying fox individual has the probability of exploring other locations.

(2) Death and Replacement of Flying Foxes

There are two causes of death in flying foxes: overheating death and suffocation. Flying foxes that are too far away from the coolest tree in the population to return and in an extremely hot area are killed by overheating and are replaced by new individuals generated by Formula (8). At the same time, a survival list (*SL*) is introduced, which includes a certain scale of unique optimal solutions currently found within the population.

(8) $$x_{i,j}^{t+1}=\frac{\sum_{k=1}^{n}{SL}_{k,j}^{t}}{n}$$

In this scenario, *n* stands for a fluctuating number between 2 and the total number of *SL* and ${SL}_{k,j}^{t}$ represents the *j*-th element of the *k*-th individual in the *SL* of the *t*-th iteration.

Under the suffocation strategy, when a flying fox individual is surrounded by other flying foxes and reaches crowding, a suffocation death operation is executed. The number *nc* of flying fox individuals with the same individual value in the whole flying fox population and the corresponding suffocation probability *pD* = (*nc* − 1)/(Population size) are calculated. A stochastic value within the interval (0,1) is obtained. If this value is less than *pD*, then two flying foxes die, and two new flying foxes are produced using Formula (8). In the other case, two flying foxes *R*_1_ and *R*_2_ are randomly selected to cross over to generate offspring to replace the dead flying foxes; the result is shown in Formula (9).

(9) $$offspring1=L*R_{1}+\left(1-L\right)*R_{2}\;offspring2=L*R_{2}+\left(1-L\right)*R_{1}$$

where *L* is a random number between (0 and 1). The offspring can only be generated through the two ways in Formula (9). The probability of selecting either Formula (8) or Formula (9) is equal at 0.5. Conversely, if the random number is greater than *pD*, then two flying fox individuals survive. If *nc* is odd, then the above operation is performed for the even number of them, stipulating that one of the remaining flying fox individuals dies and a new flying fox individual is generated by Formula (8) for replacement.

This paper is dedicated to extending the unique survival strategy in the FFO algorithm to the domain of multi-objective problems, aiming to achieve better convergence speed and search results to overcome the challenges faced by MOPs [30,31].

## 3. The Framework of MOEA/D-FFO

### 3.1. Overview

MOEA/D-FFO solves MOPs using the strategy of flying fox migration. MOEA/D in MOEA has excellent performance and wide applicability, but, unlike the operation of MOEA/D that extracts two random operators in the corresponding neighbor matrices of each operator to perform cross-variant substitution, MOEA/D-FFO treats each operator, and its corresponding neighbor matrix, as a population of flying foxes to be iterated. In addition, MOEA/D-FFO uses the penalty-based boundary intersection (PBI) [32,33] method to decompose multi-objective optimization problems. The PBI method is widely used in many decomposition-based MOEAs, and the PBI method itself can be adapted to the nature and requirements of the problem after the introduction of penalty parameters to find an optimal balance between distributivity and convergence.

In MOEA/D-FFO, all flying foxes will be evolved iteratively as a population. In contrast to the strategy of the single-objective algorithm, this paper takes each flying fox and its corresponding neighboring individuals as a subpopulation; that is to say, in the algorithm with a population size of *N*, it contains *N* subpopulations. In each of these subpopulations, an independent coolest tree, i.e., the optimal solution of the current subpopulation, is set. Within the same subpopulation, the coolest tree attracts flying foxes in that subpopulation towards it, thus avoiding overheating death. Within the whole population (i.e., between different subpopulations), a global *SL* is set for information exchange between different subpopulations.

**Figure 1:**
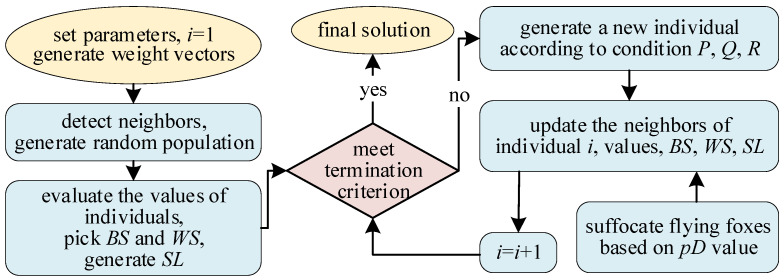
The overall framework of MOEA/D-FFO.

The general MOEA/D-FFO framework is depicted in Figure 1. For flying foxes within the population with different distances from the coolest tree, corresponding migration operations are executed according to different formulas, and, finally, suffocation judgment is used to construct new solutions. This contributes to broadening the variety within the population. During the search process, this solves the problem of poor population diversity due to the single coolest tree set within the original population.

Furthermore, in order to improve the clarity of the flying foxes’ survival strategy employed by MOEA/D-FFO, this paper uses an established taxonomy to identify its features in order to clarify how the algorithm works. Gore and Reynolds proposed an exploration-based taxonomy for emergent behavior analysis in simulations, which facilitated the process of interpreting taxonomies [34]. This taxonomy consists of three separate dimensions. When evaluating the algorithms, it identifies a certain type within each dimension and explores the algorithms in a way that is appropriate for this type, making the workings of the algorithms clearer and more explicit. Following the ternary structure of this taxonomy, MOEA/D-FFO is identified as <stochastic, unpredictable, materializing>. The results of this classification allow for effective exploration and application of the given behaviors in MOEA/D-FFO.

### 3.2. Key Components of MOEA/D-FFO

MOEA/D-FFO improves on some basic concepts and evolutionary strategies to better improve the population diversity as well as the rate of convergence exhibited by the algorithm. The important improved parts of the flying foxes’ survival strategy can be summarized in the following two aspects.

(1) Distance Calculation Method

MOEA/D’s strategy for calculating the distance between individuals is to calculate the Euclidean distance between the corresponding weight vectors of the individuals, as shown in Formula (10).

(10) $$d\left(E,F\right)=\sqrt{\sum_{i=1}^{n}{\left(e_{i}-f_{i}\right)}^{2}}$$

where *e* and *f* denote the weight vectors of individuals *E* and *F* in *n*-dimensional space, respectively. The FFO algorithm employs a system comprising six fuzzy rules to adaptively govern the value of every variable, considering the distance of each flying fox individual from the current optimal solution.

Combining the above distance calculation methods, this paper uses Formula (10) to calculate the Euclidean distance between flying foxes, thereby establishing *N* subpopulations. Additionally, the individual value is used as the location parameter of an individual, and its value is calculated by summing the product of value of each sub-objective function for an individual and the relative weight vector. Based on the value of the distance from the individual flying fox to the coolest tree (the current optimal solution), the flying foxes in the population are categorized into three types of individuals, i.e., close to the coolest tree, far from the coolest tree, and too far from the coolest tree. This improvement successfully extends the flying foxes’ survival strategy from a single-objective domain to a multi-objective domain.

(2) Iteration Method

MOEA/D-FFO adjusts the survival strategy of the flying fox algorithm and improves the offspring generation mechanism and the population update mechanism for the flying fox population to enhance the comprehensive capability of the algorithm.

On the one hand, for the offspring generation mechanism, compared with the original FFO algorithm, MOEA/D-FFO draws on the offspring generation method of MOEA/D. For the flying foxes that are too far away from the coolest tree, a new offspring is generated by randomly selecting two individuals in the corresponding neighbor matrices to mate after the death operation is performed to further enhance the searching capability of the flying fox populations.

On the other hand, for the population updating mechanism, compared with the FFO algorithm that compares and replaces the original flying fox individuals each time a child is generated, MOEA/D-FFO uses the new child to update the corresponding neighbor matrix of the corresponding flying fox individual to speed up the iteration speed of the population. Although this mechanism increases to some extent the suffocation probability of the optimal individual when the population performs the suffocation operation, the operation helps to speed up the further exploration of the PF for flying foxes located in the coolest position, thus increasing the convergence speed of the population.

### 3.3. Description and Analysis of MOEA/D-FFO

MOEA/D-FFO will select *M* weight vectors *λ*_1_, …, *λ_m_* to break down the MOP into *M* single-objective subproblems, each associated with its corresponding weight vector *λ_i_*, followed by randomly generating *N* candidate solutions for the multi-objective problem, each of which contains a generative decision variable for an individual flying fox, the objective values of the evaluated individual sub-objectives, and the constraint violations. These flying fox decision variables, i.e., the vectors labeling the positions of the *i*-th flying foxes in the whole space, are composed of a d-dimensional vector *x_i_ = (x_i_*_1_, …, *x_id_)* and evaluated by the problem objective function *f(x)*, with subsequent iterative updates and other operations. In each iteration, MOEA/D-FFO will use the parameters and variables as shown below.

*N* flying fox individual solutions: *x*_1_, …, *x_N_ ϵ Ω*, each flying fox individual contains its decision variables, the objective values of each sub-objective, and constraint violations.

The weight matrix *W_ij_* of dimension *(N*, *M)*: *i* belongs to (1, *N*) and *j* belongs to (1, *M*), which represents the weight of the flying fox individual *x_i_* on the sub-objective *j* and satisfies *W_i_*_1_ + … + *W_iM_* = 1.

*F*_1_, …, *F_N_*: *F_i_* represents the evaluated value of the flying fox individual, *Fi = f*_1_*(x_i_) × W_i_*_1_ + … + *f_M_(x_i_) × W_iM_*.

*T* is the number of neighbors: *T* is set as *N*/10 upwards to an integer; this number is chosen to strike a balance between the weight vectors so that the number of neighbors for each individual is neither too dense nor too scattered.

*B*_1_, …, *B_N_* is the neighbor matrix: *B_i_* corresponds to the *T* neighboring individuals corresponding to the flying fox individual *i*. By computing the Euclidean distance between the weight vector associated with flying fox individual *i* and the weight vectors associated with other individuals within the population, after ascending the order, starting from the first individual, *T* individuals are selected as the neighbor vectors of the flying fox individual *i*.

*BS*_1_, …, *BS_N_*: *BS_i_* represents the optimal individual in the population, corresponding to flying fox individual *i* in the population.

*WS*_1_, …, *WS_N_*: *WS_i_* represents the worst individual in the population, corresponding to flying fox individual *i* in the population.

*SL* is the survival list of the flying fox algorithm: the size of *SL* is specified to be *N*/4 rounded integers. It is used to store the first *N*/4 optimal individuals in each iteration of the entire flying fox population.

As shown in Algorithm 1, MOEA/D-FFO consists of four main steps.
**Algorithm 1.** MOEA/D-FFO Algorithm.**Input** population size, dimension, weight vectors,
 the number of the weight vectors in the neighborhood of each weight vector
**Output** final population
**Begin**1.   Initialize weight matrix *W_ij_* with dimensions *(N*, *M)*.2.   Compute Euclidean distance between individuals to generate neighbor matrix *B*_1_, …, *B_N_*.3.   For *i* = 1 to *N*:4.    Generate initial flying fox individual *x_i_*.5.    Evaluate the fitness *F_i_* of individual *x_i_*.6.   End.7.   Initialize *BS_i_*, *WS_i_,* and *SL*.8.   For *i* = 1 to *N*: #Iteration Using FFO Method.9.    Compute norm *N*_1_ as the difference between *F_i_* and *BS_i_*.10.  Compute norm *N*_2_ as the difference between *BS_i_* and *WS_i_*.11.  Determine the position of flying fox individual based on conditions *P*, *Q*, *R*.12.  If condition *P* is satisfied:13.   Randomly select two distinct individuals *x_a_*, *x_b_* from *B_i_*.14.   Generate a new individual *y* through *x_a_* and *x_b_*.15.  Elseif condition *Q* is satisfied:16.   Generate a new individual *y* by moving *x_i_* towards the direction of the optimal solution according to Formula (5).17.  Elseif condition *R* is satisfied:18.   Generate a new individual *y* by moving *x_i_* to another location to avoid suffocation according to Formulas (6) and (7).19.  End. 20.  Update *B_i_* and relevant information in the population.21.  Perform suffocation judgment for flying foxes with the same fitness value. #Suffocate Flying Foxes 22.  If the randomly generated number is less than the suffocation probability *pD*:23.   If the number of flying foxes gathered in the same area is even,24.    generate 2 new flying fox individuals for replacement via Formula (8) or (9), with the probability of 0.5 for each method.25.   Else26.    perform the above operation on an even number of foxes, declare the remaining fox dead, and replace it with a new flying fox generated using Formula (8). 27.   End.28.  End.29.  Update relevant parameters in the population.30. End.31. If the maximum iteration count has not been reached: #Termination.32.  Return to Line 8 and continue iteration.33. Else34.  Terminate iteration and return the final result.35. End.**End**

Step 1 Generation of Neighbors and Population: Firstly, a weight matrix *W* with dimension *(N*, *M)* is generated, and the Euclidean distance of the weight vectors between individuals is calculated to generate the neighbor matrix *B*. For *i =* 1, …, *N*, generate the initial flying fox individual *x_i_* and evaluate the individual value *F_i_* of *x_i_*. After the initial population generation, detect the optimal solution and the worst solution of each flying fox individual and its corresponding neighbors, and initialize the group of optimized solutions *BS* and the group of worst solutions *WS* for each population. Finally, filter the whole population and initialize *SL* for subsequent operations.

Step 2 Iteration Using FFO Method: For *i =* 1, …, *N*, calculate the number of paradigms of the difference between *F_i_* and *BS_i_* of individual *x_i_* of flying foxes, denoted as *N*_1_. And, *N*_2_ is the number of paradigms of the difference between *BS_i_* and *WS_i_*. Use Formula (11) to determine the flying fox individual *x_i_* in its corresponding population position, where both *a* and *b* are parameters that are set between 0 and 1.

(11) $$condition\;P:\;N_{1}>b\times N_{2}\;condition\;Q:\;a\times N_{2}<N_{1}<b\times N_{2}\;condition\;R:\;N_{1}<a\times N_{2}$$

Condition *P* means that the flying foxes are too far away from the coolest tree in the population and cannot reach it. Condition *Q* means that the flying foxes are far away from the coolest tree in the population but can reach it through migration. Condition *R* means that the flying foxes are too close to the coolest tree in the population and can easily be suffocated and die.

If condition *P* is satisfied, two different individuals *x_a_* and *x_b_* are randomly selected from *B_i_*. *x_a_* and *x_b_* are simulated through a crossover inheritance operation to generate offspring, thus obtaining a new individual *y*. If individual *x_i_* of the flying fox satisfies condition *Q*, then Formula (5) is used to make individual *x_i_* of the flying fox move towards the optimal solution in the corresponding population, generating a new individual *y*. If condition *R* is satisfied, then Formulas (6) and (7) are used to make the flying fox move to other positions, generating a new individual *y* and avoiding the flying fox being too close to the optimal individual, which may lead to suffocation.

At the end of the iteration, the corresponding parameters and neighbor matrices in the flying foxes’ population are updated.

Step 3 Suffocating Flying Foxes: For the population after iteration, *i* = 1, 2, …, *N*, in the overall flying fox population, find the individuals with the same value as the optimal individual in the current subpopulation and perform suffocation operation. If the randomly generated number is less than the calculated suffocation probability *pD*, then perform the suffocation operation, and generate two new flying fox individuals for replacement via Formula (8) or (9), with a probability of 0.5 for each method. And, if the number of flying fox individuals clustered in the same area is odd, perform the above operation for an even number of them, stipulate that one of the remaining flying foxes is dead, and provide for the death of one of the remaining flying fox individuals via Formula (8) to generate a new flying fox individual for replacement. At the end of the suffocation, the corresponding parameters in the flying fox population are updated.

Step 4 Termination: After the whole population completes migration and suffocation, verify if the maximum iteration limit has been reached. If not, return to Step 2 to continue the iteration process. If it is reached, terminate the iteration and return the final result.

Referring to Algorithm 1, the time complexity associated with MOEA/D-FFO mainly depends on the three steps in each iteration process, i.e., domain selection, maintenance of the external population, and generation of children. Overall, its computational complexity is *O(mTN) + O(mN*^2^*) + O(mTN*^2^*) = O(mTN*^2^*)*. *m* is the number of objectives, *T* is the domain size, and *N* is the population size. Therefore, the time complexity of the algorithm proposed in this section is similar to that of MOEA/D. However, there may be a slight increase in execution time due to the sequential execution of the child generation process of MOEA/D-FFO.

## 4. Simulation Experiments and Analysis of Results

To validate whether the flying foxes’ survival strategy in the FFO algorithm can show its excellent performance in the multi-objective domain as it does in the single-objective domain, we compare and analyze MOEA/D-FFO against five other algorithms. NSGA-II [22], proposed by Deb et al., is a high-performance multi-objective evolutionary algorithm that searches for non-dominated solutions on the PF by efficiently performing the non-dominated ordering and maintaining population diversity. In the following experiments, NSGA-II was used as one of the algorithms for comparison, considering that after improving the FFO algorithm using the decomposition-based approach, the FFO algorithm may be improved again using the non-dominated sorting approach. In addition, considering that the capability of MOEA/D-FFO may vary across different test problems, we also chose IBEA [23], which is well-adapted to different problem domains, as part of the comparison algorithms. Finally, the original MOEA/D algorithm, as well as its two improved versions, namely, Dynamic Thompson Sampling for MOEA/D (MOEA/D-DYTS) [35] and MOEA/D with updating when required (MOEA/D-UR) [36], were also included as comparison algorithms to verify whether the application of the flying foxes’ survival strategy can improve the performance compared to the original algorithm and its latest improved variants.

### 4.1. Experimental Setup

The experiments were not only conducted for the improved MOEA/D-FFO on the two test function sets but also to test how the performance of MOEA/D-FFO varies under different population sizes by continuously increasing the population size. In this experiment, three sets of population sizes (*N*) were set: 250, 500, and 750. In order to make the comparison fairer, the MOEA/D as well as the MOEA/D framework in MOEA/D-FFO were parameterized consistently with the PBI decomposition method. The maximum number of function evaluations (maxFE) for all algorithms was 200,000. All algorithms were implemented using the MATLAB-based PlatEMO platform [37]. The specific parameter settings of each algorithm are shown in Table 1. All experiments were carried out on a computer with 16 GB of RAM and a 3.20 GHz 8-core AMD Ryzen 7 5800H processor with the Windows 11 operating system installed.

In Table 1, *M* is the number of objectives, *D* is the number of decision variables, *pa* represents the probability constant, both *a* and *b* are parameters that are set between 0 and 1, and α is a positive attraction constant. In addition, the remaining parameters, neighborhood size (*T*), probability of selecting population (*δ*), update threshold (*C*), maximum number of solutions replaced by each offspring (*nr*), number of divisions of the objective space (*k*) and *kappa*, had their values extracted from the common values used in the literature.

### 4.2. Performance Metrics

In the experiment, inverted generational distance (IGD) [38] and hypervolume metric (HV) [39] were used to assess the algorithm’s effectiveness.

IGD is defined as the average distance from the Pareto optimal solution set, P*, which is true and uniformly distributed, to the solution set *P* obtained through the algorithm, as specified in Formula (12).

(12) $$IGD=\frac{\sum_{v\epsilon P^{*}}d(v,P)}{P^{*}}$$

The minimum Euclidean distance from an individual to the individual *v* in a population *P* is denoted by *d*(*v*,*P*). A certain number of individuals is uniformly selected on the real PF and represented by P*. The optimal solution set obtained by the algorithm is denoted by *P*. IGD is an evaluation index of the algorithm’s total capability, which reflects the algorithm’s distributivity and convergence. The smaller the IGD value, the better the distribution and convergence of the optimized solution set derived by the algorithm.

HV quantifies the volume occupied by the objective space region, which is delineated by the non-dominated solution set and the reference point, as derived from the multi-objective optimization algorithm. The expression for hypervolume is illustrated in Formula (13).

(13) $$HV=\delta (\cup_{i=1}^{\left|S\right|}v_{i})$$

Here, *δ* denotes the Lebesgue measure, utilized to assess volume. The notation *|S|* denotes the quantity of non-dominated sets of solutions, while *v_i_* is defined as the hypercube formed by the reference point z* in conjunction with the *i*-th solution within that set. The metric HV acts as a reliable one-dimensional quality indicator, displaying strict monotonicity with respect to Pareto domination. Higher HV values denote superior performance of the respective algorithms.

All experimental data are derived from 30 independent runs, with the mean and standard deviation meticulously recorded. These experimental outcomes underwent statistical scrutiny utilizing the Wilcoxon rank-sum test [40], applying a significance level of 0.05. The symbols +, −, and = indicate statistical superiority, inferiority, and equivalence, respectively, compared to the MOEA/D-FFO algorithm.

### 4.3. Experimental Results and Analysis

The algorithm and other compared algorithms were tested on DTLZ [41] and ZDT [42]. For better comparison, all of the algorithm result data are analyzed based on three different population sizes (250, 500, and 750) to investigate the performance of the algorithms and other comparative algorithms under various parameter settings. The best result in each row is emphasized in black. In each result, the first line of data represents the mean, the data in brackets in the second line represents the standard deviation, and the symbol (+/−/=) after the bracket in the second line indicates the result of the Wilcoxon rank-sum test. In addition, in each experimental result figure, the red circles represent the population distribution obtained by independently running the algorithm 30 times and taking the average value, the blue line represents the PF of the test problem.

(1) For a population size of 250, Table 2 and Table 3 display the experimental outcomes for all evaluated algorithms, detailing the average and standard deviation for both IGD and HV metrics.

As can be seen in Table 2, for the IGD metric, MOEA/D-FFO achieved the best results on 9 out of 12 problems. For the DTLZ test suite, the proposed algorithm performs poorly only on DTLZ4 and DTLZ7. For all other test problems in DTLZ, the proposed algorithm achieves significant advantages. This demonstrates that the population update strategy proposed in this paper is able to make the algorithm jump out of the local optimum in most cases. For the ZDT test suite, the algorithm proposed in this paper also achieves the best results. Compared to MOEA/D-UR, it performs well on four problems and slightly worse on one problem. Compared to the other four compared algorithms, MOEA/D-FFO achieves a significant advantage in all of them.

Similar results were achieved for the mean and standard deviation of HV in Table 3. MOEA/D-FFO achieved 8 best results on 12 problems, further validating the effectiveness of the proposed algorithm as well as the population updating strategy.

**Figure 2:**
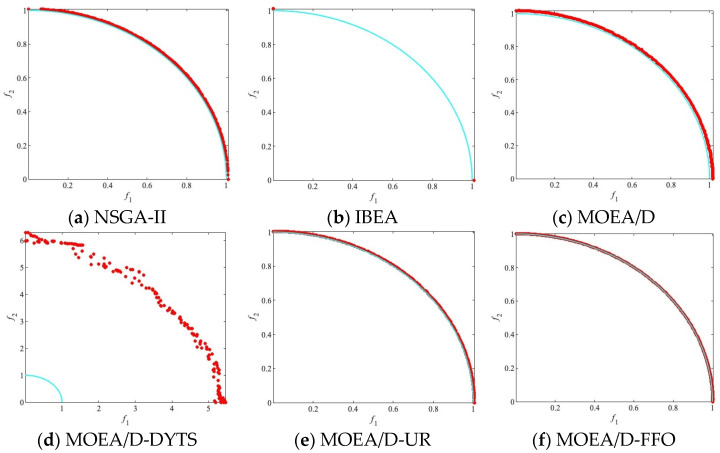
The populations of all algorithms on DTLZ3 with the median IGD values among 30 runs when N = 250.

Figure 2 illustrates the population distribution of these algorithms on PF. It can be seen that neither IBEA nor MOEA/D-DYTS converges to PF on DTLZ3, whereas among the remaining four algorithms that converge to PF, MOEA/D-FFO shows good diversity by having a more even and closer population distribution on PF.

Compared to the more homogeneous iterative approach used by some algorithms, in MOEA/D-FFO, after dividing the population into *N* small populations of flying foxes, the individuals of flying foxes in each of the small populations migrate according to the distance from the coolest tree currently found within the population. For the individuals that are farther away from the coolest tree, they will move in the direction of the coolest tree. This approach speeds up the search of the algorithm, and the convergence of the algorithm is accelerated by the introduction of the penalty mechanism of FFO, i.e., it performs suffocation operations for the individuals that are clustered near the coolest tree within the population and regenerates the individuals to replace them, thus preventing the population from falling into a locally optimal solution so that the operators that are close to the coolest tree will move in the other direction.

Moreover, MOEA/D-FFO improves the penalty mechanism for overheating death of flying foxes that are too far away from the coolest tree within the population to MOEA/D’s original way of generating offspring by using the neighbor matrix, which improves the survivability of the peripheral flying foxes and increases the possibility of the flying foxes searching for other solutions. At the same time, the mechanism of suffocation operation will inhibit the flying foxes from falling into the local optimum and make sure they search in other directions, ensuring that the final operators are not too close to each other but are spread evenly over the PF, resulting in a more homogeneous and higher-quality solution being found. This updating method is effective for the vast majority of test problems, which demonstrates the efficacy of the population updating strategy proposed in this study.

(2) For a population size of 500, Table 4 and Table 5 display the experimental outcomes for all evaluated algorithms, detailing the average and standard deviation for both IGD and HV metrics.

As revealed by Table 4 and Table 5, the proposed algorithm achieves 8 best results on 12 problems according to the IGD metric. This is similar to the performance at a population size of 250. And, compared to the HV metrics, the proposed algorithm achieves 9 best results on 12 problems, which is a better result than at a population size of 250.

Specifically, after the population size is raised to 500, the performance of MOEA/D-FFO compared to NSGA-II, IBEA, and MOEA/D-DYTS shows little difference relative to the performance at a population size of 250. For the same number of evaluations, the IGD has smaller values and MOEA/D-FFO converges faster. From the results of DTLZ1, DTLZ3, and ZDT1, it can be seen that the population distribution of MOEA/D-FFO is significantly closer to the PF compared to the other five compared algorithms. Based on the results from the HV metric, it is evident that MOEA/D-FFO demonstrates a faster convergence speed and better PF coverage compared to NSGA-II, IBEA, and MOEA/D-DYTS, especially MOEA/D-UR, after increasing the population size. This indicates that the multi-objective flying foxes’ survival strategy employed by MOEA/D-FFO is more intelligent in handling larger-scale problems. In contrast to the iterative strategy that solely relies on the neighbor matrix for the generating operator, the various migration methods and the penalty mechanism used by MOEA/D-FFO constitute a more efficient population management strategy. This approach enhances global search capabilities, enabling quicker identification of the PF. These results demonstrate the capability of the proposed algorithm to tackle MOPs at a population size of 500.

**Figure 3:**
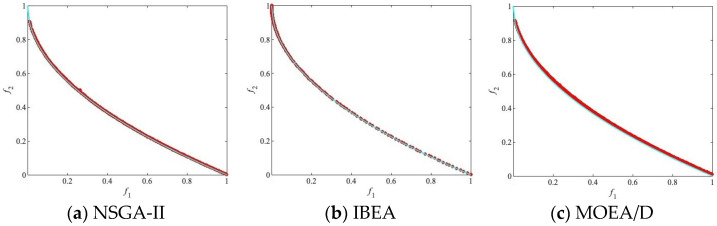

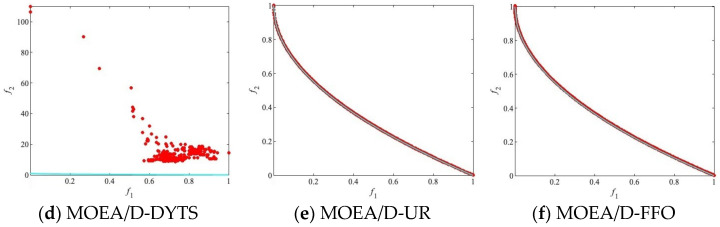
The populations of all algorithms on ZDT4 with the median IGD values among 30 runs when N = 500.

Figure 3 illustrates the population distribution of these algorithms on the PF. It is observed that MOEA/D-DYTS does not converge to the PF on ZDT4. Among the remaining five algorithms that do converge to the PF, MOEA/D-FFO and MOEA/D-UR exhibit a more uniform population distribution on the PF, demonstrating good diversity.

(3) For a population size of 750, Table 6 and Table 7 display the experimental outcomes for all evaluated algorithms, detailing the average and standard deviation for both IGD and HV metrics.

At a population size of 750, it is demonstrated by Table 6 that MOEA/D-FFO achieves the 10 best results on 12 problems in terms of IGD metrics. Specifically, compared to the other compared algorithms, it performs poorly only on the DTLZ7 test function, while it achieves significant advantages on the other problems. Compared to the experimental results at population sizes of 250 and 500, it is easy to see that the comprehensive capability of the algorithms proposed in this paper is optimal on the test functions at all population sizes up to 750. Therefore, concerning larger population sizes, MOEA/D-FFO can still achieve a performance advantage due to the same number of evaluations resulting in fewer iterations.

As can be seen from Table 7, in terms of HV metrics, compared to MOEA/D-UR, MOEA/D-FFO performs slightly worse on the test functions DTLZ5 and DTLZ7, stays flat on DTLZ2, DTLZ4, ZDT3, and ZDT4, and performs better on the remaining six test functions. The capability advantage of MOEA/D-FFO is more obvious compared to the other four compared algorithms. It can be seen that when the population size is 750, although the number of iterations is reduced, the performance of MOEA/D-FFO does not decrease and maintains more advantages in HV metrics.

From these experimental results, it can be seen that the population size increase does not lead to the performance degradation of MOEA/D-FFO, and it even performs better in some test problems, such as DTLZ4, DTLZ6, ZDT2, ZDT4, and so on.

**Figure 4:**
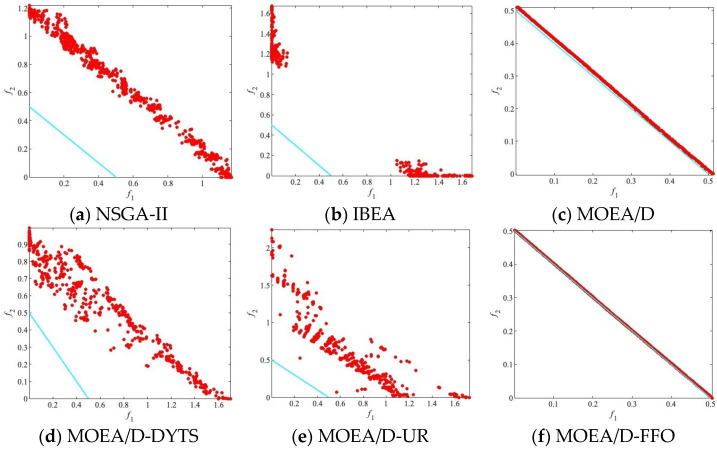
The populations of all algorithms on DTLZ1 with the median IGD values among 30 runs when N = 750.

Figure 4 illustrates the population distribution of these algorithms on PF. It can be seen that on DTLZ1, NSGA-II, IBEA, MOEA/D-DYTS, and MOEA/D-UR do not converge to PF. Meanwhile, for MOEA/D and MOEA/D-FFO that converge to PF, MOEA/D-FFO shows good diversity with a more homogeneous and closer population distribution on PF.

In summary, this section validates the performance of MOEA/D-FFO on two multi-objective test benchmark problems under different parameter settings. The experimental outcomes demonstrate that MOEA/D-FFO can achieve good performance under different population size settings (i.e., different number of iterations). Furthermore, as the population size increases, the number of iterations decreases for the same number of evaluations, yet MOEA/D-FFO still achieves satisfactory performance. This also shows that compared with other compared algorithms, the proposed algorithm is not sensitive to the population size, is more adaptable, and has a better application prospect in real-world applications. In conclusion, from the experimental results of IGD metrics and HV metrics, MOEA/D-FFO can take the lead in most of the problems, which indicates that the flying fox migration strategy and the population update strategy in this paper have excellent performance in solving MOPs.

### 4.4. Real-World Applications

The research in this paper focuses on solving three real-world optimization problems that are important in different fields. One of them is the multiline distance minimization problem (ML-DMP) [43], which involves computing the Euclidean distance from a point to a group of straight lines. These straight lines are usually considered to be edges passing through a given square polygon, which itself may have a varying number of vertices. By optimizing these distance values, we can obtain more efficient solutions in various application scenarios, such as path planning in a geographic information system (GIS) or obstacle avoidance in robot navigation.

The multi-objective knapsack problem (MOKP) [44] is often described as selecting different items in a knapsack of finite capacity to maximize or minimize the value of multiple objective functions. Unlike the traditional knapsack problem, which has only one objective function (usually maximizing the total value or minimizing the total weight), the multi-objective knapsack problem involves considering multiple objectives simultaneously. As a result, the multi-objective knapsack problem is more tough to solve.

In addition, the multi-objective next release problem (MONRP) [45] is a complex challenge involving software engineering and requirements management. It is designed to help software product managers determine which features to include in the next software release to maximize customer satisfaction and, at the same time, keep organizational costs within reason. Due to the NP-hard problem, this problem not only requires in-depth algorithms and optimization methods but also needs to fully take the competition in the market and the dynamics of customer needs into account.

In the experiments, NSGA-II, IBEA, MOEA/D, MOEA/D-DYTS, and MOEA/D-UR are still chosen as the compared algorithms, with the same parameter settings as in Section 4.1. The number of evaluations is 10,000. The means and standard deviations of the HV metric obtained from 30 independent runs are shown in Table 8. Because the PFs of these three real-world optimization problems are unknown, HV is used as the only evaluation metric. Based on the experimental findings, it is evident that the MOEA/D-FFO algorithm can find a solution and achieves the best results on all three problems. In summary, the proposed algorithm has excellent performance on these real-world applications as well.

## 5. Conclusion and Future Work

The NFL theorem illustrates that there is no single algorithm that can efficiently solve all problems, which is even more evident for MOPs that need to balance multiple conflicting objectives. Therefore, new optimization algorithms are constantly being proposed to apply to specific problems or to enhance the capability bottlenecks of the algorithms. In this paper, we propose a decomposition-based multi-objective flying foxes optimization algorithm (MOEA/D-FFO). It uses MOEA/D as a framework to apply the survival strategy of the FFO algorithm to tackle MOPs. Aiming to extend the basic FFO algorithm to a multi-objective algorithm, this paper designs and implements a strategy to compute the positions of flying fox individuals. In order to realize a good population management scheme, a new mechanism for child generation is introduced, and a new population update mechanism is proposed to enhance the search efficacy and convergence speed of the flying fox population to improve the algorithm’s overall capability. In this paper, we compare and analyze MOEA/D-FFO as well as five other algorithms using two classical test function sets, DTLZ and ZDT. When the population size is set to 250, 500, and 750, MOEA/D-FFO shows excellent performance on both IGD and HV, with an overall better level than the compared algorithms. In addition, it is also focused on solving three real-world optimization problems, all of which deliver satisfactory outcomes.

Although the algorithm proposed in this paper has excellent performance and performs well in solving MOPs, it still has room for improvement. In the future, the algorithm proposed in this paper can be extended. On the one hand, the fuzzy auto-adjustment method can be applied to MOEA/D-FFO to avoid the problematic experimental results due to the manual setting of parameters by introducing fuzzy logic and dynamically adjusting the setup parameters in MOEA/D-FFO. On the other hand, the application of FFO’s survival strategy can also be upgraded from the multi-objective domain to the many-objective domain, further exploring its greater potential, such as the method for judging the distances in the population of flying foxes, the method for classifying the subpopulations and the migratory strategies of the individuals of flying foxes that have different distances from the coolest tree, etc.

## Figures and Tables

**Table 1:** Parameter settings of each algorithm.

Algorithm	Parameter Settings
NSGA-II	*M*: 2, *D*: 30
IBEA	*M*: 2, *D*: 30, *kappa*: 0.05
MOEA/D	*M*: 2, *D*: 30, *T*: *N*/10
MOEA/D-DYTS	*M*: 2, *D*: 30, *T*: 20, *δ*: 0.8, *C*: 100
MOEA/D-UR	*M*: 2, *D*: 30, *T*: *N*/10, *δ*: 0.9, *nr*: 2, *k*: 10
MOEA/D-FFO	*M*: 2, *D*: 30, *T*: *N*/10, *pa*: 0.5, *a*: 0.14, *b*: 0.15, α: 0.5

**Table 2:** The means and standard deviations of the IGD metric derived through MOEA/D-FFO and five compared MOEAs on two test suites when N = 250.

Problem	NSGA-II	IBEA	MOEA/D	MOEA/D-DYTS	MOEA/D-UR	MOEA/D-FFO
DTLZ1	4.3949 × 10^−3^ (1.64 × 10^−3^) −	4.4554 × 10^−2^ (5.75 × 10^−3^) −	6.3607 × 10^−3^ (2.24 × 10^−3^) −	3.3548 × 10^+0^ (5.63 × 10^+0^) −	3.2315 × 10^−3^ (9.71 × 10^−4^) −	**1.1545 × 10^−3^** **(6.62 × 10^−4^)**
DTLZ2	1.9805 × 10^−3^ (3.24 × 10^−5^) −	9.3430 × 10^−3^ (1.03 × 10^−3^) −	1.5778 × 10^−3^ (2.57 × 10^−6^) =	1.6134 × 10^−3^ (1.64 × 10^−5^) −	1.6343 × 10^−3^ (1.93 × 10^−5^) −	**1.5769 × 10^−3^** **(5.11 × 10^−9^)**
DTLZ3	1.1258 × 10^−2^ (4.25 × 10^−3^) −	3.4497 × 10^−1^ (1.47 × 10^−3^) −	1.5842 × 10^−2^ (4.87 × 10^−3^) −	1.1390 × 10^+1^ (2.30 × 10^+1^) −	8.1295 × 10^−3^ (5.51 × 10^−3^) −	**4.0807 × 10^−3^** **(3.19 × 10^−3^)**
DTLZ4	2.2979 × 10^−3^ (9.19 × 10^−4^) +	1.5680 × 10^−1^ (3.08 × 10^−1^) −	7.5877 × 10^−2^ (2.34 × 10^−1^) =	**1.6824 × 10^−3^** **(5.19 × 10^−5^) **+	1.6966 × 10^−3^ (5.20 × 10^−5^) +	1.4968 × 10^−1^ (3.12 × 10^−1^)
DTLZ5	1.9932 × 10^−3^ (3.99 × 10^−5^) −	9.5602 × 10^−3^ (6.90 × 10^−4^) −	1.5769 × 10^−3^ (4.66 × 10^−9^) −	1.6139 × 10^−3^ (1.63 × 10^−5^) −	1.6233 × 10^−3^ (1.12 × 10^−5^) −	**1.5769 × 10^−3^** **(2.58 × 10^−9^)**
DTLZ6	2.2329 × 10^−3^ (7.85 × 10^−5^) −	2.5237 × 10^−2^ (3.55 × 10^−3^) −	1.5769 × 10^−3^ (1.52 × 10^−9^) =	1.5773 × 10^−3^ (2.20 × 10^−7^) −	1.6384 × 10^−3^ (3.94 × 10^−5^) −	**1.5769 × 10^−3^** **(2.65 × 10^−9^)**
DTLZ7	3.7365 × 10^−3^ (1.77 × 10^−3^) +	4.3078 × 10^−3^ (1.85 × 10^−3^) +	2.6950 × 10^−1^ (2.27 × 10^−1^) =	1.3454 × 10^−1^ (2.12 × 10^−1^) +	**2.4144 × 10^−3^** **(2.75 × 10^−5^) **+	2.6784 × 10^−1^ (2.28 × 10^−1^)
ZDT1	1.8257 × 10^−3^ (2.33 × 10^−5^) −	1.6717 × 10^−3^ (2.00 × 10^−5^) −	1.7671 × 10^−3^ (1.32 × 10^−5^) −	1.5691 × 10^−3^ (1.80 × 10^−5^) =	1.6279 × 10^−3^ (8.40 × 10^−5^) −	**1.5616 × 10^−3^** **(6.15 × 10^−6^)**
ZDT2	1.8909 × 10^−3^ (3.13 × 10^−5^) −	4.7491 × 10^−3^ (3.56 × 10^−4^) −	1.5210 × 10^−3^ (3.00 × 10^−6^) =	1.5213 × 10^−3^ (6.06 × 10^−6^) =	1.5307 × 10^−3^ (8.79 × 10^−6^) −	**1.5198 × 10^−3^** **(4.95 × 10^−6^)**
ZDT3	3.8152 × 10^−3^ (1.74 × 10^−3^) −	1.3896 × 10^−2^ (2.44 × 10^−3^) −	1.6583 × 10^−1^ (5.59 × 10^−2^) −	4.1696 × 10^−3^ (6.17 × 10^−6^) −	**3.0425 × 10^−3^** **(3.22 × 10^−5^) **+	3.6849 × 10^−3^ (5.24 × 10^−5^)
ZDT4	6.0091 × 10^−3^ (3.92 × 10^−3^) =	1.3898 × 10^−1^ (1.95 × 10^−1^) −	2.4720 × 10^−2^ (2.60 × 10^−2^) −	1.5404 × 10^+0^ (9.64 × 10^−1^) −	2.8860 × 10^−3^ (6.29 × 10^−4^) =	**2.8176 × 10^−3^** **(6.07 × 10^−4^)**
ZDT6	1.5022 × 10^−3^ (2.81 × 10^−5^) −	1.7212 × 10^−3^ (2.27 × 10^−5^) −	3.3377 × 10^−3^ (4.36 × 10^−4^) −	1.2345 × 10^−3^ (1.44 × 10^−6^) −	1.4563 × 10^−3^ (2.44 × 10^−5^) −	**1.1209 × 10^−3^** **(3.17 × 10^−4^)**
+/−/=	2/9/1	1/11/0	0/7/5	2/8/2	3/8/1	—

**Table 3:** The means and standard deviations of the HV metric derived through MOEA/D-FFO and five compared MOEAs on two test suites when N = 250.

Problem	NSGA-II	IBEA	MOEA/D	MOEA/D-DYTS	MOEA/D-UR	MOEA/D-FFO
DTLZ1	5.7470 × 10^−1^ (4.05 × 10^−3^) −	4.7704 × 10^−1^ (1.27 × 10^−2^) −	5.6995 × 10^−1^ (5.47 × 10^−3^) −	3.4149 × 10^−1^ (2.58 × 10^−1^) −	5.7735 × 10^−1^ (2.62 × 10^−3^) −	**5.8336 × 10^−1^** **(1.86 × 10^−3^)**
DTLZ2	3.4923 × 10^−1^ (3.53 × 10^−5^) −	3.4883 × 10^−1^ (1.27 × 10^−4^) −	3.4943 × 10^−1^ (3.65 × 10^−7^) −	3.4926 × 10^−1^ (2.83 × 10^−5^) −	3.4920 × 10^−1^ (4.25 × 10^−4^) −	**3.4943 × 10^−1^** **(1.69 × 10^−7^)**
DTLZ3	3.3522 × 10^−1^ (5.81 × 10^−3^) −	1.5494 × 10^−1^ (8.94 × 10^−3^) −	3.2893 × 10^−1^ (6.70 × 10^−3^) −	1.2442 × 10^−1^ (1.62 × 10^−1^) −	3.3913 × 10^−1^ (7.67 × 10^−3^) −	**3.4539 × 10^−1^** **(4.82 × 10^−3^)**
DTLZ4	3.4897 × 10^−1^ (7.59e × 10^−4^) +	2.9695 × 10^−1^ (1.09 × 10^−1^) −	3.2334 × 10^−1^ (8.17 × 10^−2^) +	3.4917 × 10^−1^ (4.59 × 10^−5^) +	**3.4931 × 10^−1^** **(2.44 × 10^−4^) **+	2.9773 × 10^−1^ (1.09 × 10^−1^)
DTLZ5	3.4924 × 10^−1^ (3.33 × 10^−5^) −	3.4881 × 10^−1^ (9.17 × 10^−5^) −	3.4943 × 10^−1^ (2.51 × 10^−7^) =	3.4924 × 10^−1^ (2.64 × 10^−5^) −	3.4926 × 10^−1^ (2.25 × 10^−4^) =	**3.4943 × 10^−1^** **(8.56 × 10^−8^)**
DTLZ6	3.4914 × 10^−1^ (5.32 × 10^−5^) −	3.4513 × 10^−1^ (6.94 × 10^−4^) −	3.4943 × 10^−1^ (2.59 × 10^−8^) =	3.4943 × 10^−1^ (1.41 × 10^−7^) −	**3.4948 × 10^−1^** **(1.77 × 10^−5^) **+	3.4943 × 10^−1^ (8.60 × 10^−8^)
DTLZ7	2.4306 × 10^−1^ (4.01 × 10^−4^) +	2.4299 × 10^−1^ (3.97 × 10^−4^) +	2.0225 × 10^−1^ (3.40 × 10^−2^) =	2.2316 × 10^−1^ (3.25 × 10^−2^) +	**2.4339 × 10^−1^** **(6.18 × 10^−6^) **+	2.0289 × 10^−1^ (3.47 × 10^−2^)
ZDT1	7.2258 × 10^−1^ (3.15 × 10^−5^) −	7.2277 × 10^−1^ (1.52 × 10^−5^) −	7.2172 × 10^−1^ (4.82 × 10^−5^) −	7.2268 × 10^−1^ (8.62 × 10^−5^) −	7.2284 × 10^−1^ (7.03 × 10^−5^) −	**7.2373 × 10^−1^** **(2.83 × 10^−5^)**
ZDT2	4.4714 × 10^−1^ (3.83 × 10^−5^) −	4.4685 × 10^−1^ (5.88 × 10^−5^) −	4.4738 × 10^−1^ (2.82 × 10^−5^) =	4.4735 × 10^−1^ (2.00 × 10^−5^) =	4.4739 × 10^−1^ (1.35 × 10^−4^) =	**4.4739 × 10^−1^** **(3.71 × 10^−5^)**
ZDT3	5.9994 × 10^−1^ (6.17 × 10^−4^) −	5.9868 × 10^−1^ (5.71 × 10^−4^) −	6.2788 × 10^−1^ (5.21 × 10^−2^) −	5.9990 × 10^−1^ (2.42 × 10^−5^) −	6.0031 × 10^−1^ (8.10 × 10^−6^) −	**6.9820 × 10^−1^** **(6.90 × 10^−5^)**
ZDT4	7.1559 × 10^−1^ (5.78 × 10^−3^) =	6.3134 × 10^−1^ (1.24 × 10^−1^) −	6.8898 × 10^−1^ (3.76 × 10^−2^) −	2.5191 × 10^−2^ (7.23 × 10^−2^) −	**7.2022 × 10^−1^** **(9.19 × 10^−4^) **+	7.1905 × 10^−1^ (1.04 × 10^−3^)
ZDT6	3.9012 × 10^−1^ (6.24 × 10^−5^) −	3.9014 × 10^−1^ (5.87 × 10^−5^) −	3.8656 × 10^−1^ (7.03 × 10^−4^) −	3.9071 × 10^−1^ (1.31 × 10^−5^) −	3.9008 × 10^−1^ (1.48 × 10^−4^) −	**3.9689 × 10^−1^** **(4.95 × 10^−4^)**
+/−/=	2/9/1	1/11/0	1/7/4	2/9/1	4/6/2	—

**Table 4:** The means and standard deviations of the IGD metric derived through MOEA/D-FFO and five compared MOEAs on two test suites when N = 500.

Problem	NSGA-II	IBEA	MOEA/D	MOEA/D-DYTS	MOEA/D-UR	MOEA/D-FFO
DTLZ1	4.5115 × 10^−2^ (1.11 × 10^−1^) −	7.2762 × 10^−2^ (2.85 × 10^−2^) −	7.6007 × 10^−3^ (1.99 × 10^−3^) −	7.9618 × 10^+0^ (1.07 × 10^+1^) −	4.4420 × 10^−2^ (1.15 × 10^−1^) −	**1.6661 × 10^−3^** **(8.26 × 10^−4^)**
DTLZ2	1.0037 × 10^−3^ (1.01 × 10^−5^) −	6.7262 × 10^−3^ (4.91 × 10^−4^) −	**7.9902 × 10^−4^** **(4.59 × 10^−6^) **=	8.4767 × 10^−4^ (1.60 × 10^−5^) −	8.8908 × 10^−4^ (1.39 × 10^−5^) −	8.0176 × 10^−4^ (4.67 × 10^−6^)
DTLZ3	2.9141 × 10^−2^ (2.19 × 10^−2^) −	3.5452 × 10^−1^ (8.07 × 10^−3^) −	1.8411 × 10^−2^ (5.03 × 10^−3^) −	1.2269 × 10^+0^ (1.97 × 10^+0^) −	2.1115 × 10^−2^ (4.68 × 10^−3^) −	**4.9953 × 10^−3^** **(1.83 × 10^−3^)**
DTLZ4	9.8777 × 10^−4^ (1.35 × 10^−5^) −	6.4972 × 10^−3^ (4.55 × 10^−4^) −	1.4906 × 10^−1^ (3.13 × 10^−1^) −	8.7837 × 10^−4^ (2.40 × 10^−5^) −	8.9820 × 10^−4^ (2.42 × 10^−5^) −	**7.9647 × 10^−4^** **(7.56 × 10^−6^)**
DTLZ5	9.8587 × 10^−4^ (1.84 × 10^−5^) −	6.7747 × 10^−3^ (2.42 × 10^−4^) −	8.0211 × 10^−4^ (6.35 × 10^−6^) =	8.4117 × 10^−4^ (1.30 × 10^−5^) −	8.9832 × 10^−4^ (2.16 × 10^−5^) −	**8.0023 × 10^−4^** **(6.51 × 10^−6^)**
DTLZ6	1.1063 × 10^−3^ (1.95 × 10^−5^) −	2.8097 × 10^−2^ (2.50 × 10^−3^) −	1.0593 × 10^−3^ (8.61 × 10^−4^) −	7.8772 × 10^−4^ (6.71 × 10^−7^) −	8.8588 × 10^−4^ (1.20 × 10^−5^) −	**7.8691 × 10^−4^** **(6.22 × 10^−9^)**
DTLZ7	**1.0273 × 10^−3^** **(1.52 × 10^−5^) **+	1.3327 × 10^−3^ (7.51 × 10^−5^) +	3.9994 × 10^−1^ (1.40 × 10^−1^) =	8.9503 × 10^−2^ (1.86 × 10^−1^) +	1.3202 × 10^−3^ (2.89 × 10^−5^) +	4.0011 × 10^−1^ (1.40 × 10^−1^)
ZDT1	9.0726 × 10^−4^ (1.84 × 10^−5^) −	8.4264 × 10^−4^ (6.71 × 10^−6^) −	1.0668 × 10^−3^ (2.03 × 10^−5^) −	8.7852 × 10^−4^ (7.05 × 10^−5^) −	9.0406 × 10^−4^ (2.16 × 10^−5^) −	**8.0667 × 10^−4^** **(1.38 × 10^−5^)**
ZDT2	9.4069 × 10^−4^ (1.19 × 10^−5^) −	3.1937 × 10^−3^ (1.53 × 10^−4^) −	**7.6499 × 10^−4^** **(3.99 × 10^−6^) **=	8.2350 × 10^−4^ (1.04 × 10^−4^) −	8.1171 × 10^−4^ (7.94 × 10^−6^) −	7.6954 × 10^−4^ (1.13 × 10^−5^)
ZDT3	1.0430 × 10^−3^ (1.22 × 10^−5^) =	9.8817 × 10^−3^ (3.53 × 10^−4^) −	4.0094 × 10^−3^ (2.87 × 10^−4^) −	2.1133 × 10^−3^ (1.86 × 10^−5^) −	1.7249 × 10^−3^ (4.94 × 10^−5^) −	**1.0392 × 10^−3^** **(2.01 × 10^−5^)**
ZDT4	2.7576 × 10^−3^ (8.55 × 10^−4^) −	4.3180 × 10^−3^ (3.79 × 10^−4^) −	8.8488 × 10^−3^ (1.96 × 10^−3^) −	9.5438 × 10^+0^ (4.54 × 10^+0^) −	1.9384 × 10^−3^ (3.34 × 10^−4^) =	**1.8994 × 10^−3^** **(1.00 × 10^−3^)**
ZDT6	1.7023 × 10^−3^ (1.66 × 10^−4^) +	9.3604 × 10^−4^ (2.29 × 10^−5^) +	4.1974 × 10^−3^ (7.46 × 10^−4^) =	**6.5427 × 10^−4^** **(1.18 × 10^−4^) **+	1.5122 × 10^−3^ (1.09 × 10^−4^) +	3.6210 × 10^−3^ (6.27 × 10^−4^)
+/−/=	2/9/1	2/10/0	0/7/5	2/10/0	2/9/1	—

**Table 5:** The means and standard deviations of the HV metric derived through MOEA/D-FFO and five compared MOEAs on two test suites when N = 500.

Problem	NSGA-II	IBEA	MOEA/D	MOEA/D-DYTS	MOEA/D-UR	MOEA/D-FFO
DTLZ1	5.0578 × 10^−1^ (1.73 × 10^−1^) −	3.9785 × 10^−1^ (7.25 × 10^−2^) −	5.6706 × 10^−1^ (5.14 × 10^−3^) −	1.0151 × 10^−1^ (2.16 × 10^−1^) −	5.0838 × 10^−1^ (1.79 × 10^−1^) −	**5.8209 × 10^−1^** **(2.17 × 10^−3^)**
DTLZ2	3.5007 × 10^−1^ (1.59 × 10^−5^) −	3.4973 × 10^−1^ (4.72 × 10^−5^) −	3.5014 × 10^−1^ (4.24 × 10^−6^) −	3.5002 × 10^−1^ (1.86 × 10^−5^) −	3.4995 × 10^−1^ (2.32 × 10^−4^) =	**3.5015 × 10^−1^** **(1.34 × 10^−6^)**
DTLZ3	3.1096 × 10^−1^ (3.12 × 10^−2^) −	1.0883 × 10^−1^ (3.62 × 10^−2^) −	3.2533 × 10^−1^ (7.40 × 10^−3^) −	1.9758 × 10^−1^ (1.54 × 10^−1^) −	3.2164 × 10^−1^ (6.42 × 10^−3^) −	**3.4438 × 10^−1^** **(2.60 × 10^−3^)**
DTLZ4	3.5007 × 10^−1^ (1.01 × 10^−5^) −	3.4976 × 10^−1^ (4.60 × 10^−5^) −	2.9830 × 10^−1^ (1.09 × 10^−1^) −	3.4997 × 10^−1^ (2.17 × 10^−5^) −	3.5003 × 10^−1^ (2.25 × 10^−4^) =	**3.5016 × 10^−1^** **(9.08 × 10^−6^)**
DTLZ5	3.5006 × 10^−1^ (2.23 × 10^−5^) −	3.4973 × 10^−1^ (2.50 × 10^−5^) −	3.5014 × 10^−1^ (5.81 × 10^−6^) −	3.5003 × 10^−1^ (1.89 × 10^−5^) −	3.5000 × 10^−1^ (2.65 × 10^−4^) =	**3.5015 × 10^−1^** **(2.79 × 10^−6^)**
DTLZ6	3.5002 × 10^−1^ (1.99 × 10^−5^) −	3.4512 × 10^−1^ (6.94 × 10^−4^) −	3.4973 × 10^−1^ (1.39 × 10^−3^) =	3.5017 × 10^−1^ (1.21 × 10^−7^) −	3.5001 × 10^−1^ (7.50 × 10^−5^) −	**3.5017 × 10^−1^** **(7.87 × 10^−8^)**
DTLZ7	**2.4367 × 10^−1^** **(3.14 × 10^−6^) **+	2.4362 × 10^−1^ (9.38 × 10^−6^) +	1.8280 × 10^−1^ (2.13 × 10^−2^) =	2.3007 × 10^−1^ (2.84 × 10^−2^) +	2.4359 × 10^−1^ (9.91 × 10^−5^) +	1.8279 × 10^−1^ (2.13 × 10^−2^)
ZDT1	7.2357 × 10^−1^ (1.67 × 10^−5^) −	7.2365 × 10^−1^ (5.76 × 10^−6^) −	7.2241 × 10^−1^ (4.63 × 10^−5^) −	7.2335 × 10^−1^ (1.5 × 10^−4^) −	7.2357 × 10^−1^ (5.82 × 10^−5^) −	**7.2440 × 10^−1^** **(3.17 × 10^−5^)**
ZDT2	4.4809 × 10^−1^ (1.12 × 10^−5^) −	4.4786 × 10^−1^ (2.29 × 10^−5^) −	**4.4815 × 10^−1^** **(3.07 × 10^−5^) **=	4.4797 × 10^−1^ (2.86 × 10^−4^) −	4.4811 × 10^−1^ (1.09 × 10^−4^) =	4.4813 × 10^−1^ (5.63 × 10^−5^)
ZDT3	6.0087 × 10^−1^ (3.52 × 10^−6^) =	5.9960 × 10^−1^ (4.40 × 10^−5^) −	5.9903 × 10^−1^ (1.12 × 10^−4^) −	6.0052 × 10^−1^ (4.47 × 10^−5^) −	6.0070 × 10^−1^ (1.76 × 10^−4^) −	**6.0089 × 10^−1^** **(9.01 × 10^−5^)**
ZDT4	7.2056 × 10^−1^ (1.12 × 10^−3^) +	7.1926 × 10^−1^ (7.94 × 10^−4^) =	7.1172 × 10^−1^ (2.47 × 10^−3^) −	0.0000 × 10^+0^ (0.00 × 10^+0^) −	**7.2165 × 10^−1^** **(4.60 × 10^−4^) **+	7.1947 × 10^−1^ (1.51 × 10^−3^)
ZDT6	3.8940 × 10^−1^ (2.24 × 10^−4^) −	3.9090 × 10^−1^ (4.19 × 10^−5^) −	3.8578 × 10^−1^ (8.95 × 10^−4^) −	3.9125 × 10^−1^ (1.82 × 10^−4^) −	3.8960 × 10^−1^ (1.69 × 10^−4^) −	**3.9657 × 10^−1^** **(8.34 × 10^−4^)**
+/−/=	2/9/1	1/10/1	0/9/3	1/11/0	2/6/4	—

**Table 6:** The means and standard deviations of the IGD metric derived through MOEA/D-FFO and five compared MOEAs on two test suites when N = 750.

Problem	NSGA-II	IBEA	MOEA/D	MOEA/D-DYTS	MOEA/D-UR	MOEA/D-FFO
DTLZ1	5.2250 × 10^−1^ (3.08 × 10^−1^) −	7.3502 × 10^−1^ (3.24 × 10^−1^) −	7.8214 × 10^−3^ (9.78 × 10^−4^) −	8.0414 × 10^+0^ (1.32 × 10^+1^) −	3.6377 × 10^−1^ (3.19 × 10^−1^) −	**2.1424 × 10^−3^** **(1.49 × 10^−3^)**
DTLZ2	6.6256 × 10^−4^ (6.57 × 10^−6^) −	5.6815 × 10^−3^ (3.14 × 10^−4^) −	5.7002 × 10^−4^ (1.31 × 10^−5^) =	6.0817 × 10^−4^ (2.25 × 10^−5^) −	6.1478 × 10^−4^ (4.26 × 10^−6^) −	**5.6842 × 10^−4^** **(1.10 × 10^−5^)**
DTLZ3	1.0471 × 10^+0^ (1.01 × 10^+0^) −	1.9615 × 10^+0^ (1.47 × 10^+0^) −	1.9432 × 10^−2^ (3.87 × 10^−3^) −	2.0449 × 10^+0^ (4.44 × 10^+0^) −	1.0836 × 10^+0^ (8.94 × 10^−1^) −	**6.3471 × 10^−3^** **(2.19 × 10^−3^)**
DTLZ4	6.6057 × 10^−4^ (1.06 × 10^−5^) −	5.7920 × 10^−3^ (3.06 × 10^−4^) −	7.4713 × 10^−2^ (2.34 × 10^−1^) −	6.6802 × 10^−4^ (4.73 × 10^−5^) −	6.2551 × 10^−4^ (5.13 × 10^−6^) −	**5.4813 × 10^−4^** **(2.47 × 10^−5^)**
DTLZ5	6.6127 × 10^−4^ (1.37 × 10^−5^) −	5.7915 × 10^−3^ (2.92 × 10^−4^) −	9.4264 × 10^−4^ (1.18 × 10^−3^) −	6.1172 × 10^−4^ (2.32 × 10^−5^) −	6.1588 × 10^−4^ (4.16 × 10^−6^) −	**5.7505 × 10^−4^** **(1.28 × 10^−5^)**
DTLZ6	7.3660 × 10^−4^ (1.10 × 10^−5^) −	2.8897 × 10^−2^ (2.86 × 10^−3^) −	6.3783 × 10^−4^ (3.59 × 10^−4^) −	5.2518 × 10^−4^ (5.73 × 10^−7^) −	6.9883 × 10^−4^ (1.05 × 10^−4^) −	**5.2429 × 10^−4^** **(2.35 × 10^−8^)**
DTLZ7	**6.8034 × 10^−4^** **(9.33 × 10^−6^) **+	9.2440 × 10^−4^ (5.78 × 10^−5^) +	4.4447 × 10^−1^ (1.20 × 10^−4^) −	1.3332 × 10^−1^ (2.13 × 10^−1^) +	1.0113 × 10^−3^ (3.03 × 10^−5^) +	3.5654 × 10^−1^ (1.85 × 10^−1^)
ZDT1	6.0056 × 10^−4^ (8.90 × 10^−6^) −	5.6553 × 10^−4^ (5.43 × 10^−6^) =	8.2819 × 10^−4^ (2.33 × 10^−5^) −	1.0014 × 10^−3^ (4.20 × 10^−4^) −	6.7923 × 10^−4^ (2.61 × 10^−5^) −	**5.3627 × 10^−4^** **(2.66 × 10^−5^)**
ZDT2	6.1802 × 10^−4^ (9.29 × 10^−6^) −	2.4874 × 10^−3^ (1.06 × 10^−4^) −	5.3325 × 10^−4^ (2.06 × 10^−5^) =	7.1403 × 10^−4^ (2.70 × 10^−4^) −	6.0349 × 10^−4^ (1.29 × 10^−5^) −	**5.2885 × 10^−4^** **(8.10 × 10^−6^)**
ZDT3	6.9056 × 10^−4^ (5.67 × 10^−6^) −	9.6070 × 10^−3^ (2.47 × 10^−4^) −	3.2855 × 10^−3^ (3.61 × 10^−5^) −	1.4292 × 10^−3^ (1.73 × 10^−5^) −	1.4051 × 10^−3^ (4.23 × 10^−5^) −	**3.3399 × 10^−4^** **(1.83 × 10^−4^)**
ZDT4	3.2406 × 10^−3^ (9.94 × 10^−4^) =	1.6212 × 10^−2^ (1.86 × 10^−2^) −	7.5500 × 10^−3^ (1.67 × 10^−3^) −	1.6528 × 10^+1^ (4.19e × 10^+0^) −	**2.9070 × 10^−3^** **(8.27 × 10^−4^) **=	3.0880 × 10^−3^ (1.14 × 10^−3^)
ZDT6	3.7112 × 10^−3^ (4.23 × 10^−4^) −	1.9096 × 10^−3^ (3.09 × 10^−4^) −	4.6904 × 10^−3^ (3.75 × 10^−4^) −	1.2997 × 10^−3^ (1.54 × 10^−3^) =	2.8214 × 10^−3^ (2.70 × 10^−4^) −	**1.2882 × 10^−3^** **(4.54 × 10^−4^)**
+/−/=	1/10/1	1/10/1	0/10/2	1/10/1	1/10/1	—

**Table 7:** The means and standard deviations of the HV metric derived through MOEA/D-FFO and five compared MOEAs on two test suites when N = 750.

Problem	NSGA-II	IBEA	MOEA/D	MOEA/D-DYTS	MOEA/D-UR	MOEA/D-FFO
DTLZ1	6.4075 × 10^−2^ (1.39 × 10^−1^) −	0.0000 × 10^+0^ (0.00 × 10^+0^) −	5.6662 × 10^−1^ (2.42 × 10^−3^) −	1.4324 × 10^−1^ (1.83 × 10^−1^) −	1.6636 × 10^−1^ (2.14 × 10^−1^) −	**5.8095 × 10^−1^** **(3.91 × 10^−3^)**
DTLZ2	3.5033 × 10^−1^ (1.29 × 10^−5^) −	3.5005 × 10^−1^ (2.97 × 10^−5^) −	3.5032 × 10^−1^ (1.41 × 10^−5^) −	3.5027 × 10^−1^ (1.84 × 10^−5^) −	**3.5037 × 10^−1^** **(2.90 × 10^−5^) **=	3.5035 × 10^−1^ (9.30 × 10^−6^)
DTLZ3	3.8404 × 10^−2^ (6.11 × 10^−2^) −	3.7559 × 10^−3^ (1.19 × 10^−2^) −	3.2418 × 10^−1^ (5.30 × 10^−3^) −	2.1673 × 10^−1^ (1.52 × 10^−1^) −	6.9319 × 10^−2^ (1.08 × 10^−1^) −	**3.4263 × 10^−1^** **(2.76 × 10^−3^)**
DTLZ4	3.5033 × 10^−1^ (1.28 × 10^−5^) −	3.5005 × 10^−1^ (2.66 × 10^−5^) −	3.2439 × 10^−1^ (8.20 × 10^−2^) −	3.5022 × 10^−1^ (2.15 × 10^−5^) −	3.5036 × 10^−1^ (3.31 × 10^−5^) =	**3.5038 × 10^−1^** **(3.22 × 10^−5^)**
DTLZ5	3.5033 × 10^−1^ (9.90 × 10^−6^) =	3.5004 × 10^−1^ (2.36 × 10^−5^) −	3.4977 × 10^−1^ (1.71 × 10^−3^) −	3.5026 × 10^−1^ (2.36 × 10^−5^) −	**3.5037 × 10^−1^** **(2.88 × 10^−5^) **+	3.5034 × 10^−1^ (6.69 × 10^−6^)
DTLZ6	3.5032 × 10^−1^ (9.21 × 10^−6^) −	3.4512 × 10^−1^ (7.11 × 10^−4^) −	3.5022 × 10^−1^ (6.34 × 10^−4^) −	3.5042 × 10^−1^ (1.72 × 10^−7^) −	3.5027 × 10^−1^ (1.09 × 10^−4^) −	**3.5042 × 10^−1^** **(3.37 × 10^−8^)**
DTLZ7	**2.4374 × 10^−1^** **(2.14 × 10^−6^) **+	2.4370 × 10^−1^ (5.65 × 10^−6^) +	1.7604 × 10^−1^ (1.16 × 10^−5^) =	2.2337 × 10^−1^ (3.26 × 10^−2^) +	2.4356 × 10^−1^ (1.20 × 10^−4^) +	1.8918 × 10^−1^ (2.77 × 10^−2^)
ZDT1	7.2388 × 10^−1^ (9.13 × 10^−6^) −	7.2394 × 10^−1^ (4.76 × 10^−6^) −	7.2267 × 10^−1^ (5.10 × 10^−5^) −	7.2308 × 10^−1^ (6.10 × 10^−4^) −	7.2380 × 10^−1^ (4.15 × 10^−5^) −	**7.2465 × 10^−1^** **(4.64 × 10^−5^)**
ZDT2	4.4841 × 10^−1^ (7.52 × 10^−6^) =	4.4823 × 10^−1^ (1.29 × 10^−5^) −	4.4835 × 10^−1^ (7.29 × 10^−5^) −	4.4800 × 10^−1^ (5.92 × 10^−4^) −	4.4832 × 10^−1^ (7.59 × 10^−5^) −	**4.4846 × 10^−1^** **(3.42 × 10^−5^)**
ZDT3	6.0097 × 10^−1^ (1.49 × 10^−6^) =	5.9968 × 10^−1^ (3.81 × 10^−5^) −	5.9923 × 10^−1^ (1.28 × 10^−4^) −	6.0020 × 10^−1^ (6.66 × 10^−5^) −	6.0087 × 10^−1^ (1.50 × 10^−4^) =	**6.0097 × 10^−1^** **(1.58 × 10^−3^)**
ZDT4	7.2004 × 10^−1^ (1.29 × 10^−3^) =	7.0952 × 10^−1^ (1.23 × 10^−2^) −	7.1315 × 10^−1^ (2.42 × 10^−3^) −	0.0000 × 10^+0^ (0.00 × 10^+0^) −	7.2030 × 10^−1^ (1.15 × 10^−3^) =	**7.2035 × 10^−1^** **(1.54 × 10^−3^)**
ZDT6	3.8684 × 10^−1^ (5.53 × 10^−4^) −	3.8910 × 10^−1^ (4.47 × 10^−4^) −	3.8540 × 10^−1^ (5.29 × 10^−4^) −	3.9041 × 10^−1^ (1.88 × 10^−3^) −	3.8786 × 10^−1^ (3.83 × 10^−4^) −	**3.9627 × 10^−1^** **(5.81 × 10^−4^)**
+/−/=	1/7/4	1/11/0	0/11/1	1/11/0	2/6/4	—

**Table 8:** HV means and standard deviations of all algorithms for 30 independent runs on the three real-world problems.

Problem	NSGA-II	IBEA	MOEA/D	MOEA/D-DYTS	MOEA/D-UR	MOEA/D-FFO
ML-DMP	0.0000 × 10^+0^ (0.00 × 10^+0^) −	1.5584 × 10^−3^ (2.20 × 10^−4^) −	5.4466 × 10^−3^ (9.48 × 10^−4^) −	2.7837 × 10^−3^ (4.08 × 10^−4^) −	2.7366 × 10^−5^ (5.77 × 10^−5^) −	**6.3532 × 10^−3^** **(1.92 × 10^−4^)**
MOKP	5.3348 × 10^−1^ (3.08 × 10^−3^) −	5.3271 × 10^−1^ (1.91 × 10^−3^) −	5.1329 × 10^−1^ (2.59 × 10^−3^) −	5.3072 × 10^−1^ (1.11 × 10^−3^) −	5.2305 × 10^−1^ (2.38 × 10^−3^) −	**5.4076 × 10^−1^** **(2.68 × 10^−3^)**
MONRP	6.5120 × 10^−1^ (9.65 × 10^−3^) −	6.5919 × 10^−1^ (8.08 × 10^−3^) −	1.8750 × 10^−1^ (3.91 × 10^−3^) −	6.2678 × 10^−1^ (6.50 × 10^−3^) −	6.1283 × 10^−1^ (5.51 × 10^−3^) −	**6.8428 × 10^−1^** **(1.01 × 10^−2^)**
+/−/=	0/3/0	0/3/0	0/3/0	0/3/0	0/3/0	—

## Data Availability

The original contributions presented in this study are included in this article; further inquiries can be directed to the corresponding authors.
